# Immunohistochemical detection of the 1,25-dihydroxy vitamin D receptor in the human vagina

**Published:** 2014-12

**Authors:** Tae-Hee Kim, Hae-Hyeog Lee, Junsik Park

**Affiliations:** *Department of Obstetrics and Gynecology, Soonchunhyang University College of Medicine, Bucheon, 420-767, Republic of Korea.*

**Keywords:** *Epithelium*, *Estrogens*, *Ezrin*, *Receptor*, *Vagina*, *Vitamin D*

## Abstract

**Background:**

Vitamin D plays a critical role in the regulation of growth and differentiation of squamous epithelium. The pleiotropic effects of 1,25-dihydroxyvitamin D_3_ [1,25(OH)_2_D_3_], include proliferative, pro-apoptotic, and pro-differentiation effects on numerous cell types. Although 1,25-dihydroxyvitamin D_3 _is involved in the regulation and differentiation of epithelium, there is no data in the aspect of the distribution of 1,25-(OH)_2_D_3_ receptor (VDR), estrogen receptor-β (ER-β) and ezrin, and how it changes according to the menstrual phases and serum vitamin D level in the human vagina.

**Objective:**

To detect of the 1,25-dihydroxyvitamin D_3_ receptor (VDR), estrogen receptor-β (ER-β), and ezrin in human vagina.

**Materials and Methods:**

This cross-sectional study enrolled 15 premenopausal women who had hysterectomies. Vaginal tissues were obtained from vaginal vaults of hysterectomized uterus and processed for immunohistochemical analysis. We recorded the localization, distribution, and expression of VDR, ER-β, and ezrin in the vaginal epithelium.

**Results:**

VDR was detected in all layers of vaginal epithelium regardless of the menstrual cycle stage and serum 25-hydroxyvitamin D levels.

**Conclusion:**

In this study, we have characterized the presence and distribution of VDR, ER-β, and ezrin in human vaginal epithelium, including menstrual cycle-related and vitamin D-related expression. However, the VDR-related mechanisms underlying vaginal epithelial proliferation and differentiation remain to be elucidated.

## Introduction

Vitamin D plays a critical role in the regulation of growth and differentiation of squamous epithelium (1, 2). The biological effects of 1,25(OH)_2_D_3_ may be regulated by an intracellular receptor of the 1,25(OH)_2_D_3_ receptor (VDR), which is in the steroid thyroid nuclear receptor superfamily (3). The human vagina has a tubular architecture composed of a hormone-dependent, cyclically self-replacing, and keratinizing epithelial layer (4). The mechanism underlying changes in the vagina is not clearly understood. 

Only in rats, there was a study demonstrating that 1,25(OH)_2_D_3_ induced proliferation of vaginal epithelium and up-regulated VDR expression in the vaginal epithelium (5). Moreover, it was proposed that the moesin-ezrin-radixin-merlin (MERM) family of proteins is key element in the vagina (6). Especially, it has been reported that estrogen regulates expression, activation, and function of ezrin and moesin (-). Given these observations, we suggest that VDR, ER, and ezrin have major roles in the vagina. 

We hypothesized that vitamin D receptor (VDR), estrogen receptor (ER) and ezrin expression may be associated with vaginal proliferation and differentiation during the menstrual cycle. We conducted an immunohistochemical analysis of human vaginal epithelium to determine the relationship between VDR, ER-β, and ezrin. We confirmed expression of VDR, ER-β, and ezrin in the human vagina and correlated changes during the menstrual phases with serum vitamin D levels.

## Materials and methods

**Tissue Collection and Preparation**

This cross-sectional study enrolled fifteen premenopausal women who had hysterectomies from May 2011 to November 2011 at Soonchunhyang University Bucheon Hospital. Vaginal tissues were obtained from vaginal vaults of hysterectomized uterus. The Soonchunhyang University Bucheon hospital Institutional Review Board (IRB) approved the study (SCHBC_IRB_10-88). Fully informed consent was obtained from the patients, who had hysterectomies for uterine myoma or uterine adenomyosis. Our exclusion criteria were: patients who had used hormone replacement therapy or digitalis or any other medication likely to affect the vaginal epithelium. For measurement of 25-hydroxyvitamin D_3_ [25 (OH) D_3_] in serum, we obtained peripheral blood from subjects prior to operation.

In the operating room, vaginal tissues were taken immediately after removal of the uterus from a cervical vault site that was considered to be macroscopically representative of the vagina. The obtained vaginal tissues were immediately sent to Soonchunhyang Medical Research Laboratory (Bucheon, Republic of Korea). Vaginal tissues were chilled and stored at -80^o^C until they were used. Paraffin sections from each specimen were stained with hematoxylin and eosin to examine the full-thickness vagina.

**Immunohistochemistry**

All vaginal tissues were fixed in 10% formaldehyde solution, embedded in paraffin blocks, and then cut into 5-µm thick sections. Five-micron tissue sections were collected on poly-L-lysine-coated slides (Sigma-Aldrich Corp., St. Louis, MO, USA). Each tissue section was deparaffinized in xylene and rehydrated through a graded ethanol series (10). Thereafter, slides were washed in phosphate-buffered saline (PBS; pH 7.4) three times for 5 minutes. For antigen retrieval, slides were heated in a microwave oven at 600^o^C with 0.01 M sodium citrate buffer (pH 6.0) for 20 min. After 1 hr cooling at room temperature, the slides were washed three times for 5 min in PBS. Endogenous peroxidases were blocked by treating the sections with 0.3% hydrogen peroxide (Fisher ChemAlert Guide, New Jersey, USA) for 30 min, followed by washing three times with PBS. 

The slides were then incubated in a humidified chamber with blocking buffer (5% bovine serum +0.5% tween-20 in PBS) for 1 hr at room temperature. Primary antibody was applied in a moist chamber overnight at 4^o^C. Polyclonal antibodies were raised against VDR, ER-β, and ezrin. The antibodies used were rabbit anti-VDR primary polyclonal antibody (ab3508, 1:2000 dilution, Abcam Inc., Cambridge, MA, USA), rabbit anti-ER-β, primary polyclonal antibody (ab3577, 1:500, Abcam Inc., Cambridge, MA, USA), and mouse anti-ezrin primary monoclonal antibody (ab4069, 1:100, Abcam Inc., Cambridge, MA). 

After three additional rinsing steps with PBS for 5 min, biotinylated universal antibody (anti-rabbit/mouse IgG) was added for 1 hr, and the samples were treated with Vectastain avidin-biotin complex (ABC) kit for 30 min. After being washed with PBS, the sections were developed using diaminobenzidine (DAB; Vector Laboratories Inc., Burlingame, CA) for 1 min at room temperature. Counterstaining was performed with Mayer’s hematoxylin (Merck, Darmstadt, Germany). All experiments were executed with control staining without the primary antibody to ensure that negative controls remained unstained.

**Phase of menstrual cycle**

Menstrual cycle phases were determined based on the patient’s last menstrual period (LMP) and histopathological reports. Patients were classified by menstrual cycle as secretory phase or proliferative phase.

**Relative density scoring and statistical analysis**

All slides were evaluated by a pathologist according to an arbitrary semi-quantitative reference scale depending on the intensity of immunoreactive cells in individual layers of the vagina: 0= negative, 1= weak, 2= moderate, and 3= strong. A cell was considered “stained” if there was a discrete brown color associated with it that was different from the nuclei of cells stained only with hematoxylin or above any black ground color. The relative intensities of immunostained images were de convoluted to isolated DAB signals based on counterstaining, and the data were quantified using Image J (Version: software 1.45X; National Institutes of Health, Bethesda, Maryland, MD, USA). 

**Patient’s consent**

Written informed consent was obtained from the patient for publication of this original article.

**Statistical analysis**

We input data with Excel (Microsoft 2007 Office) and performed statistical analyses using Statistical Package for the Social Sciences (SPSS) Version 12.0 (SPSS Inc, Chicago, IL, USA). The semi-quantitative data were considered to be non-parametric; therefore, they were analyzed using the Mann-Whitney U-test. When the relative intensities of immunostained images were compared, statistical significance was analyzed using Student’s *t*-test. Results of both tests were considered statistically significant if p<0.05.

## Results

Human vaginal epithelium consists of a basal layer, suprabasal layer, and superficial layer (apical cells, cornified cells); we observed expression in each layer. Immunoreactive-VDR, -ER-β, and -ezrin were present in all layers of the vagina. Light microscopic examinations showed that immunohistochemical staining for ER-β was more intense in the suprabasal and basal layers than the superficial layers. Strong ezrin immunostaining was observed through the vaginal epithelium. 

**Figure 1:**
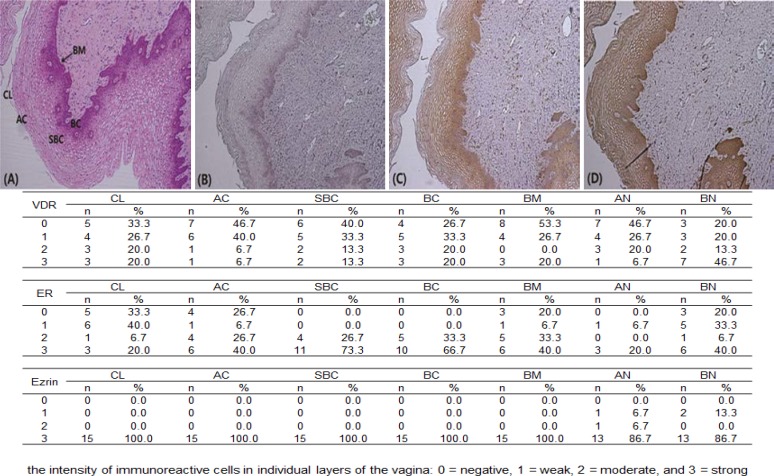
(A) H&E stain shows full layers of vaginal epithelium. (B) Immunohistochemical staining for ER-β was more intense in the suprabasal and basal layers than the superficial layers. (C, D) Immunoreactive-ezrin and- VDR were present in all layers of the vagina. (E) All slides were evaluated by a pathologist according to an arbitrary semi-quantitative reference scale, depending on the intensity of immunoreactive cells in individual layers of the vagina: 0= negative, 1= weak, 2= moderate, and 3= strong.

The ir-VDR was weakly, but definitely expressed through the vaginal epithelium. However, the basal nuclei of the vaginal epithelium demonstrated stronger immunostaining for VDR compared to the apical nuclei (Figure 1B). Therefore, ir-VDR, -ER-β, and -ezrin were expressed in the human vagina, and were more intense in the basal layer than the superficial layer, except for ir-ezrin. 

Overall, ir-ezrin was observed throughout the vaginal epithelium, but the apical nuclei demonstrated denser immunostaining than the basal nuclei. We hypothesized that the menstrual cycle and vitamin D state might affect the expression of VDR, ER-β, and ezrin in the vagina. To test our hypothesis, we investigated the enrolled patients’ menstrual phases and serum 25-hydroxyvitamin D_3_ [25(OH)D_3_] levels and compared the expression of VDR, ER- β, and ezrin in the vagina.

**Menstrual cycle and expression of VDR, ER**-β,** and Ezrin in the vagina**

**Figure 2:**
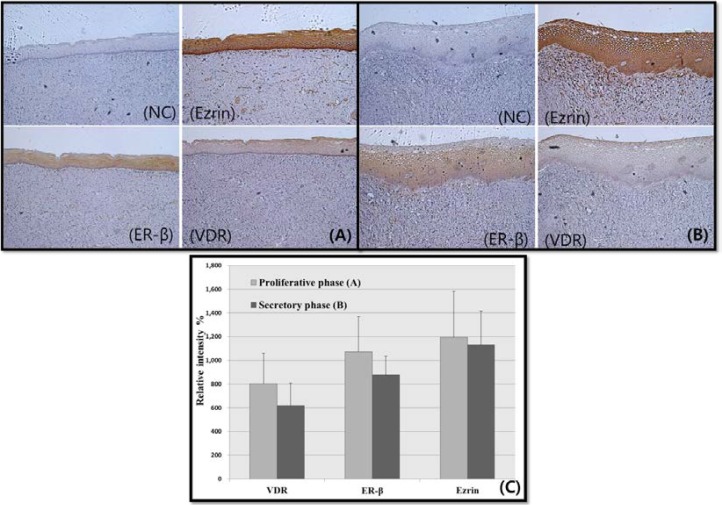
The expression of 1,25-dihydroxyvitamin D3 receptor (VDR), estrogen receptor (ER)-β, and ezrin in human vagina across the menstrual phases. (A) proliferative phase vagina (n=7) and (B) secretory phase vagina (n=8) show expression of VDR, ER-β, and ezrin. (M: 100x). Serum 25-hydroxyvitamin D3 was insufficient. (10-30 ng/mL) (C) The intensity of each signal was quantified and is represented on a bar graph. Statistical analysis of vitamin D receptor (VDR) (Mann-Whitney U-test, p= 0.908), estrogen receptor (ER)-β (Student’s t-test, p= 0.563) and ezrin expression (Mann-Whitney U-test, p= 0.817). NC= negative control.

Overall VDR, ER-β, and ezrin expression were increased during the proliferative phase (n= 7), as compared to the secretory phase (n= 8) and VDR, ER-β, and ezrin expression were found on the basal nuclei (Figure 2). However, these differences were not significant [VDR (p= 0.908), ER- β (p= 0.563), ezrin (p= 0.817)].

**Vitamin D level and expression of VDR, ER**-β,** and Ezrin in the vagina**

Because two patients refused serum 25(OH)D_3_ evaluation, we could compare vaginal tissues from 13 patients. The vitamin D levels of enrolled patients were low. The mean serum 25(OH)D_3_ level of the thirteen patients was 12.0±5.2 (SD). Nine patients were vitamin D insufficient (10-30 ng/mL) and four patients were vitamin D deficient (<10 ng/mL). The thirteen vaginal tissues were examined by microscopy and image J. 

**Figure 3:**
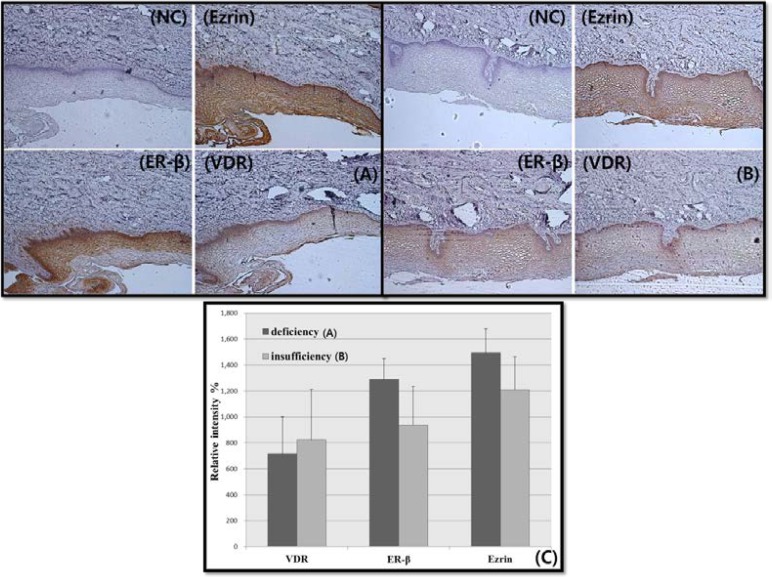
The expression of 1,25-dihydroxyvitamin D3 receptor (VDR), estrogen receptor (ER)-β, and ezrin in the human vagina and vitamin D state. (A) Vitamin D insufficiency state (<10 ng/mL), (B) Vitamin D deficiency state (10-30 ng/mL). The menstrual phases were histologically confirmed as proliferative phase. (C) The intensity of each signal was quantified and is represented in the graph. Statistical analysis of vitamin D receptor (VDR) (Mann-Whitney U-test, p= 0.537), estrogen receptor (ER)-β (Student’s t-test, p= 0.545), and ezrin expression (Mann-Whitney U-test, p= 1.000). NC= negative control.

In the insufficient group, VDR was slightly more expressed than in the deficient group, but ER-β and ezrin showed the opposite pattern. In addition, ER-β and ezrin expression were increased in vaginal tissues from the vitamin D deficient group (Figure 3), although these differences were not statistically significant [VDR (p=0.537), ER- β (p=0.545), ezrin (p=1.000)].

**CL= cornified layer **

**AC= apical cells **

**SBC= suprabasal cells**

**BC= basal cells**

**M= basal membrane**

**AN= apical nucleus **

**BN= basal nucleus**

## Discussion

In this study, we examined VDR, ER-β, and ezrin expression in human vaginal epithelium by immunohistochemical analysis. While there have been several studies of the ER in the vagina, this is the first study to investigate VDR and ezrin in human vaginal epithelium (5, 8, 9, 11, 12). We expected to identify changes in these proteins during the menstrual cycle in response to the vitamin D level. We noted that VDR, ER-β, and ezrin expression tended to increase in basal nuclei during the proliferative phase, as compared to the secretory phase. This indicates that estrogens affect VDR, ER-β, and ezrin activity in the vagina. In addition, the VDR was preferentially localized in the basal and supra-basal layers, where cells enter into the proliferation and differentiation program, and correlated with ER-β localization. This indicates that VDR, ER-β, and ezrin act together in the vagina.

Most tissues in the body have receptors for the active form of vitamin D, 1,25(OH)_2_D_3_ or calcitriol. These receptors are appropriately named VDRs and are potential targets for tissue regulation. The actions of the active form of 1,25(OH)_2_D_3_ are similar to those of other steroid hormones and are mediated by its binding to the VDR. The VDR is a member of the superfamily of nuclear hormone receptors, including receptors for steroid and thyroid hormones and retinoic acid (13). Immunostaining for VDR in the female rat has been demonstrated in ovarian follicles, granulosa cells, and the epithelium of fallopian tubes, the uterus and cervix (5). Furthermore, there was an animal study demonstrating that 1,25(OH)_2_D_3_ induced proliferation of vaginal epithelium and up-regulated VDR expression in this tissue (5). However, even though 1,25(OH)_2_D_3_ may be involved in the regulation and differentiation of epithelium, there are no human data regarding the distribution and major roles of VDR in the vagina.

The presence of ER-α expression has been detected in vaginal epithelium, muscularis, and stromal fibroblasts of the vagina and urethra using *in situ* hybridization, immunohistochemistry, and binding assays (5, 8, 14). Decreasing expression of ER-α has been reported from the cervix to the vulva, while anterior to posterior vaginal tissue decreased by the same degree (        11 , 12). However, the presence of ER-β in the vagina is not as well defined as that for ER-α. Therefore, it may be necessary to characterize the differential expression and role of ER-β in the human vagina. The MERM family of proteins is focused on vaginal actions, including cellular processes, such as motility, cortical organization, and cell proliferation (15). 

Ezrin is a representative member of the MERM family, and its actions are directly influenced by estrogen via both genomic and non-genomic pathways (7). However, no human studies have followed vaginal ezrin expression during the menstrual cycle. In this study, we characterized the presence and distribution of VDR, ER-β, and ezrin in human vaginal epithelium during the menstrual cycle and the effect of vitamin D-related expression. Although the current study was limited by the small sample size, this is the first study showing consistent expression of VDR in concert with ER-β and ezrin in the human vagina. Therefore, this study provides critical information for our understanding of the vagina, which has emerged as an important component of the aging process. The health and quality of life of menopausal women is gaining importance in the medical community and should be a priority for future studies.
